# Temporal trend of leprosy among the elderly in Brazil, 2001 – 2018

**DOI:** 10.26633/RPSP.2020.12

**Published:** 2020-02-18

**Authors:** Margarida Cristiana Napoleão Rocha, Mauricio Lisboa Nobre, Leila Posenato Garcia

**Affiliations:** 1 Postgraduate Program in Public Health University of Brasilia Brasilia Brazil Postgraduate Program in Public Health, University of Brasilia, Brasilia, Brazil.; 2 Tropical Medicine Institute Federal University of Rio Grande do Norte Rio Grande do Norte Brazil Tropical Medicine Institute, Federal University of Rio Grande do Norte, Natal, Rio Grande do Norte, Brazil.; 3 Institute of Applied Economic Research Institute of Applied Economic Research Brasilia Brazil Institute of Applied Economic Research, Brasilia, Brazil.

**Keywords:** Elderly, leprosy, epidemiology, time series studies, Brazil, Anciano, lepra, epidemiología, estudios de series temporales, Brasil., Idoso, hanseníase, epidemiologia, estudos de séries temporais, Brasil.

## Abstract

**Objective.:**

To describe the temporal trends of leprosy indicators among the elderly in Brazil in 2001 – 2018.

**Methods.:**

This was an ecological time-series study of new leprosy cases in the elderly reported to the Notifiable Diseases Information System. Prais-Winsten generalized linear regression was used to estimate temporal variations.

**Results.:**

There were 687 317 new leprosy cases in Brazil from 2001 – 2018, of which 129 214 (18.8%) were among elderly people. Overall detection rates in the elderly and of new cases with grade-2 disability showed a falling trend, with an annual percent change of -4.6% (95%CI = -5.1 to -4.0) and -3.9% (95%CI = -4.6 to -3.2). New case and new multibacillary case proportions showed an increasing trend, with an annual percent change of 2.9% (95%CI = 2.6 to 3.3) and 1.4% (95%CI = 1.0 to 1.7), respectively. Detection rates for new leprosy cases in elderly people in Brazil are decreasing, but the proportion of new cases and multibacillary cases are trending upwards.

**Conclusions.:**

New cases are shifting to older age groups, and demographic transition and immunosenescence are an influence. Inadequate reduction of grade-2 disability indicates a high risk of physical disability persists. Improved contact tracing and more effective action are needed in this age group.

## RESUMEN

**Objetivo.:**

Describir las tendencias temporales de los indicadores de lepra en la población anciana en Brasil entre 2001 y 2018.

**Métodos.:**

Estudio ecológico de series temporales de casos nuevos de lepra en ancianos reportados al Sistema de Información de Enfermedades Notificables. Se utilizó regresión lineal generalizada de Prais-Winsten para estimar las variaciones temporales.

**Resultados.:**

Entre 2001 y 2018 en Brasil hubo 687 317 casos nuevos de lepra, de los cuales 129 214 (18.8%) fueron en personas de edad avanzada. Las tasas de detección en los ancianos y los casos nuevos con discapacidad de grado 2 mostraron una tendencia descendente, con un cambio porcentual anual de –4,6% (IC 95%, –5,1 a –4,0) y –3,9% (IC 95%, – 4,6 a –3,2), respectivamente. Las proporciones de casos nuevos y de casos nuevos multibacilares mostraron una tendencia creciente, con un cambio porcentual anual de 2,9% (IC 95%, 2,6 a 3,3) y 1,4% (IC 95%, 1,0 a 1,7), respectivamente. Las tasas de detección de casos nuevos de lepra en los ancianos en Brasil están disminuyendo, pero la proporción de casos nuevos y de casos multibacilares tiende a aumentar.

**Conclusiones.:**

Los casos nuevos se están desplazando hacia los grupos de mayor edad, posiblemente debido a factores relacionados con la transición demográfica y la inmunosenescencia. La insuficiente reducción de la discapacidad de grado 2 indica que el riesgo de discapacidad física continúa siendo elevado. En este grupo etario se requiere mejorar el seguimiento de los contactos y las medidas de acción.

## RESUMO

**Objetivo.:**

Descrever as tendências temporais dos indicadores da hanseníase na população idosa do Brasil de 2001 a 2018.

**Métodos.:**

O presente estudo ecológico de séries temporais analisou os novos casos de hanseníase em idosos (60 anos ou mais) notificados ao Sistema de Informação de Agravos de Notificação (SINAN). Foi usada regressão linear generalizada de Prais-Winsten para estimar as variações temporais.

**Resultados.:**

De 2001 a 2018, houve 687 317 novos casos de hanseníase no Brasil, dos quais 129 214 (18,8%) foram em idosos. As taxas de detecção em idosos e os casos novos com grau 2 de incapacidade apresentaram tendência decrescente, com variação percentual anual de –4,6% (IC95%: –5,1 a –4,0) e –3,9% (IC95%: –4,6 a –3,2), respectivamente. Por sua vez, a tendência foi crescente para novos casos e novos casos multibacilares, com variação percentual anual de 2,9% (IC95%: 2,6 a 3,3) e 1,4% (IC95%: 1,0 a 1,7), respectivamente. As taxas de detecção de novos casos de hanseníase em idosos no Brasil estão diminuindo, mas a proporção de novos casos e de casos multibacilares tende a aumentar.

**Conclusões.:**

Os novos casos estão se deslocando para os grupos etários mais velhos, possivelmente devido a fatores relacionados com a transição demográfica e a imunossenescência. A redução insuficiente na incapacidade de grau 2 indica que o risco de incapacidade física permanece elevado. Nessa faixa etária, é necessário um melhor monitoramento dos contatos e uma ação mais efetiva.

Leprosy is a chronic infectious disease, manifested by dermatological and neurological signs and symptoms (1). According to the World Health Organization (WHO), Brazil has a high leprosy burden, given the magnitude and transcendence with which the disease affects the population (2, 3). Magnitude means high occurrence of a disease in the population, while transcendence is the existence of severe clinical and epidemiological characteristics of social relevance (1).

In 2017, Brazil registered the second highest number of new cases of leprosy worldwide (*n* = 26 875) and 92.3% of the cases in the Region of the Americas (2). Of these, 6 598 new cases were detected among the elderly, at a rate of 25.4/100 000 inhabitants (twice the overall detection rate) and 24.5% of all the new cases (4). An analysis of 591 090 new leprosy cases diagnosed in Brazil in 2001 – 2013 found an increase of 6% among the elderly (5). With regard to transcendence, Brazil in 2017 was second only to India in new cases with grade-2 disability (G2D; 2); among the elderly, the detection rate for these cases was 28.8/1 million (4).

Temporal trend analysis of indicators in elderly people with leprosy can provide a dynamic diagnosis of its occurrence, explaining its progression and the risk of physical disabilities. This data can inform hypothesis formulation and intervention planning (6).

Aging is accompanied by a reduced ability of the body to defend itself against internal and external imbalances and attacks (7). Thus, age may influence leprosy progression since the host’s immune response to *Mycobacterium leprae* determines the course and clinical forms of the disease (8). Because most cases in this age group are multibacillary (5), delayed diagnosis can assist the chain of transmission and favor the emergence of physical disability (9). This situation, in turn, impacts the social security system in Brazil, which faces the difficult mission of caring for an aging population within the context of recent, complex social security reform (10, 11).

Given the rising percentage of cases among the elderly (5) and their higher risk of physical disability (12), a better understanding of leprosy within this age group was needed. The objective of this study was to describe the temporal trends of leprosy indicators among the elderly in Brazil from 2001 – 2018.

## MATERIALS AND METHODS

This was an ecological time-series study. The units of analysis were Brazil, its five regions (North, Northeast, Southeast, South, and Midwest), and its 27 *unidades federativas* (26 states and 1 federal district; UF).

### Data sources

Secondary data were obtained from the Notifiable Diseases Information System (SINAN), operated by the Ministry of Health’s Surveillance Secretariat. Population estimates for 2001 – 2018 were produced by the Brazilian Institute of Geography and Statistics and provided by the Brazilian National Health System Information Technology Department.

### Study population

The study included all new leprosy cases in the elderly diagnosed and notified to the SINAN database from 2001 – 2018. The elderly were defined according to the WHO definition and the National Policy on Health Care for the Elderly (13) as individuals 60 years of age or more. Cases excluded were any new cases that had been misdiagnosed (and removed from the database) and new cases with G2D notified in 2007 when a change was made to the, disability grade notification code (4). The latter are being evaluated by the Ministry of Health.

### Indicators

Four indicators were identified and analyzed:

1. **Detection rate of new leprosy cases in the elderly:** new leprosy cases diagnosed in the elderly per year in Brazil divided by the estimated annual population of elderly per 100 000 inhabitants.
2. **Rate of new leprosy cases in the elderly with G2D at the time of diagnosis:** new leprosy cases with G2D diagnosed in the elderly per year in Brazil divided by the estimated annual population of elderly per 1 million inhabitants.
3. **Proportion of new leprosy cases in the elderly:** new leprosy cases in the elderly per year divided by the total new cases in the same year, expressed as a percentage.
4. **Proportion of new multibacillary cases in the elderly:** new multibacillary cases in the elderly per year divided by the total new cases in the elderly in the same year, expressed as a percentage.

### Data analysis

Prais-Winsten generalized linear regression was used to estimate temporal trends. This type of regression considers the dependence of a serial measurement on its own values at earlier times when estimating parameters, which are controlled by first-order autocorrelation. Annual percent change (APC) of the measurements was quantified and 95% confidence intervals (95%CI) were estimated using the following formula, where *b*1 is the slope of the line (14):

**Annual percent change** = [−1 + 10^b1^] × 100%

**95%CI** = [−1 + 10^b1min^] × 100%; [−1 + 10^b1max^] × 100%

Indicator trends are considered to be increasing when APC is positive, and decreasing when change is negative; both are statistically significant when APC variation does not include zero. Trend is considered to be stationary when APC is nil, i.e., there is no significant difference between its value and zero (14).

The analyses were performed with Stata® Statistical Software: Release 15 (StataCorp LLC, College Station, Texas, United States).

### Ethics

This study was approved by the Ethics Committee of the University of Brasília Faculty of Health Sciences, Certification of Submission for Ethical Appraisal 77799417.6.0000.0030, Opinion 2.411.253, issued on 2 December 2017. The secondary SINAN data did not identify individuals, so anonymity was preserved.

## RESULTS

In 2001 – 2018, a total of 687 317 new leprosy cases were diagnosed in Brazil, 129 214 (18.8%) of which occurred in elderly people. During this period, the detection rate of new elderly cases showed a decreasing trend (Table 1), from 50.3 to 25.6/100 000 inhabitants, with an APC of -4.6% (95%CI: -5.1 to -4.0%). Although all regions showed a decreasing trend (*P* < 0.05), the Southeast (APC -8.0%; 95%CI: -8.6 to -7.4%) and South (APC -7.3%; 95%CI: -8.7 to -5.8%) had statistically lower decreases than the others. In the Southeast and South regions, detection rates declined from 30.5 to 8.6/100 000 and from 19.5 to 5.8/100 000, respectively. In the other regions, detection fell most in the North (APC -4.5%; 95%CI: -5.0 to -4.0), followed by the Midwest (APC -3.6%; 95%CI: -4.4 to -2.8), and the Northeast (APC -2.6%; 95%CI: -3.4 to -1.8). Among the elderly in the North, Midwest, and Northeast regions, detection fell from 150.7 to 76.5, 122.5 to 82.4, and 69.8 to 47.2/100 000, respectively. Of note is that every year, these three regions continued to see rates higher than those of Brazil as a whole, while the other two regions had lower rates (Table 1).

**FIGURE 1.**
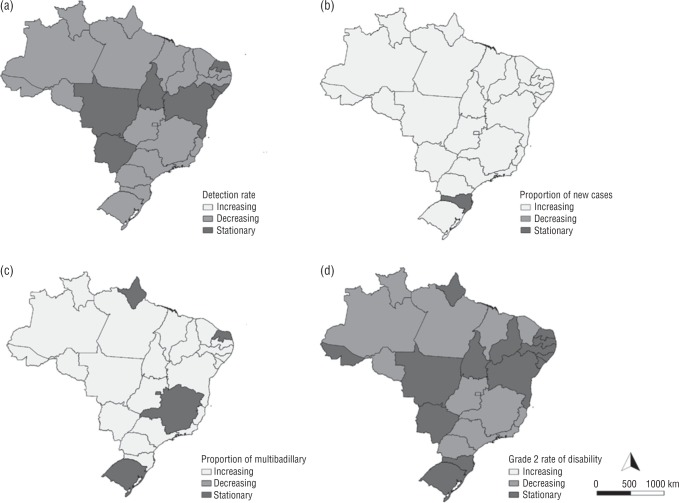
(a) Detection rate (per 100 000 inhabitants) of new leprosy cases in elderly people; (b) proportion of new leprosy cases in the elderly; (c) proportion of multibacillary new leprosy cases in the elderly; (d) rate of new leprosy cases with grade 2 physical disability at the time of diagnosis (per 1 000 000 inhabitants) in Brazil, 2001-2018

***Source:*** prepared by the authors based on data from SINAN and Brazilian Institute of Geography and Statistics (IBGE).

Among the 27 UF, the trend was stationary in 7 (*P* < 0.05) and decreasing in 20 (Table 1 and Figure 1a). Among the latter, the APC declined significantly, but less sharply, in Maranhão (-2.4%), Ceará (-3.3%), Piauí (-3.6%), and Paraíba (-4.0%); and more sharply in São Paulo (-7.2%), Paraná (-8.0%), Minas Gerais (-8.2%), Rio de Janeiro (-8.3%), Roraima (-9.5%), and Espírito Santo (-9.5%). There was no statistically significant difference in the APCs of the other 10 UF (Table 1).

**Table 1.** Detection rate (per 100 000 inhabitants) of new leprosy cases in the elderly in Brazil, its five regions, and 27 *unidades federativas* (UF), 2001–2018

Brazil/regions/UF	Rate		Annual change rate (%)	95%CI^a^		Trend
	2001	2018				
**North**	**150.7**	**76.5**	**-4.5**	**-5.0**	**-4.0**	**Decreasing**
Rondônia	187.2	83.7	-6.2	-7.8	-4.6	Decreasing
Acre	96.1	36.6	-8.7	-11.8	-5.5	Decreasing
Amazonas	92.4	21.5	-6.9	-8.4	-5.3	Decreasing
Roraima	248.3	41.3	-9.5	-11.4	-7.6	Decreasing
Pará	162.3	66.8	-5.0	-5.5	-4.4	Decreasing
Amapá	56.0	42.5	-6.4	-8.7	-4.1	Decreasing
Tocantins	193.8	254.6	0.3	-1.8	2.5	Stationary
**Northeast**	**69.8**	**47.2**	**-2.6**	**-3.4**	**-1.8**	**Decreasing**
Maranhão	173.7	116.6	-2.4	-3.3	-1.5	Decreasing
Piauí	142.3	80.2	-3.6	-4.6	-2.6	Decreasing
Ceará	94.1	51.2	-3.3	-3.9	-2.8	Decreasing
Rio Grande do Norte	21.7	16.8	-1.2	-3.5	1.2	Stationary
Paraíba	53.0	27.8	-4.0	-5.5	-2.6	Decreasing
Pernambuco	64.4	48.2	-2.8	-3.7	-1.8	Decreasing
Alagoas	20.8	24.4	-1.1	-3.1	0.9	Stationary
Sergipe	31.4	32.3	-1.2	-3.6	1.4	Stationary
Bahia	33.5	31.7	-1.3	-2.7	0.2	Stationary
**Southeast**	**30.5**	**8.6**	**-8.0**	**-8.6**	**-7.4**	**Decreasing**
Minas Gerais	34.8	10.0	-8.2	-9.1	-7.4	Decreasing
Espírito Santo	97.3	21.5	-9.5	-11.0	-8.1	Decreasing
Rio de Janeiro	39.2	11.3	-8.3	-9.1	-7.5	Decreasing
São Paulo	19.3	5.9	-7.2	-7.7	-6.6	Decreasing
**South**	**19.5**	**5.8**	**-7.3**	**-8.7**	**-5.8**	**Decreasing**
Paraná	45.2	11.1	-8.0	-9.3	-6.7	Decreasing
Santa Catarina	8.3	3.6	-6.3	-9.4	-3.1	Decreasing
Rio Grande do Sul	4.4	2.4	-6.0	-7.1	-4.8	Decreasing
**Midwest**	**122.5**	**82.4**	**-3.6**	**-4.4**	**-2.8**	**Decreasing**
Mato Grosso do Sul	70.3	31.2	-3.8	-8.3	0.8	Stationary
Mato Grosso	257.1	272.8	-0.7	-3.4	2.1	Stationary
Goiás	120.0	50.3	-6.0	-6.9	-5.0	Decreasing
Federal District	28.2	9.1	-6.2	-7.8	-4.6	Decreasing
**Brazil**	**50.3**	**25.6**	**-4.6**	**-5.1**	**-4.0**	**Decreasing**

***Source:*** prepared by the authors based on data from Sinan and Brazilian Institute of Geography and Statistics (IBGE).

a 95%CI: 95% Confidence Interval; Statistical test: Prais-Winsten generalized linear regression

The proportion of new leprosy cases among the elderly showed an increasing trend, from 16.2% in 2001 to 24.3% in 2018, with an APC of 2.9% (95%CI: 2.6 to 3.3%). This trend was similar in all regions (*P* < 0.05), but the increase was significantly greater in the North (APC 4.2%) compared to the South (2.7%), Southeast (2.8%), and Northeast (2.9%). There was no difference between the Midwest trend (3.2%) and that of the other regions. The South, Southeast, and Northeast had the highest proportions of new elderly cases during the entire time-series, increasing from 24% to 33%, 20% to 30.4%, and 17.4% to 25.8%, respectively (Table 2).

The proportion of new leprosy cases in the elderly grew significantly in all UF (*P* < 0.05), except in Santa Catarina where it was stationary. The highest APC was observed in Sergipe (4.6%), where the increase was statistically greater than in 14 other UF (Table 2 and Figure 1b).

The proportion of new multibacillary cases in the elderly also showed an increasing trend for Brazil, from 66% to 82.2% (APC 1.4%), and every region, without statistical differences among them (*P* < 0.05). During the time-series, more than 60% of new elderly cases were multibacillary in Brazil, as well as each region (Table 3).

The proportion of elderly multibacillary cases increased in 22 UF (*P* < 0.05). Mato Grosso (APC 3.3%) and Mato Grosso do Sul (3.0%) had significantly higher APCs than 11 UF, 8 of which are in the Northeast. This trend was stationary in five UF (Table 3 and Figure 1c).

The G2D rate in the elderly also showed a decreasing trend for Brazil (Table 4), falling from 50.6/1 million in 2001 to 28.3 by 2018, with a -3.9% APC (95%CI: -4.6 to -3.2). The G2D rate also fell in all of the regions. The South (APC -7.2%) and Southeast (-6.7%) showed a statistically sharper fall than the North (-2.8%), Midwest (-2.3%), and Northeast (-1.8%). The G2D rate/1 million decreased from 30.6 to 9.2 cases in the South; from 37.8 to 13.3 in the Southeast; from 157.9 to 100.0 in the North; from 93.9 to 71.1 in the Midwest; and from 56.3 to 44.4 in the Northeast. There were no differences among the three latter regions, where G2D rates were higher than for Brazil throughout the time series (Table 4).

The G2D rate among the elderly also fell in 13 UF (*P* < 0.05). Espírito Santo (APC -10.6%), Federal District (-9.4%), Espírito Santo (-10.6%), Paraná (-8.8%), and Minas Gerais (-8.4%) showed significantly greater reductions than Maranhão (-1.9%), Pará (-2.3%), Rio de Janeiro (-4.6%), São Paulo (-5.1%), and Goiás (-5.2). The trend was stationary in the remaining 14 UF, 7 of which are in the Northeast (Table 4 and Figure 1d).

## DISCUSSION

This study revealed the magnitude and transcendence trend of leprosy among the elderly in Brazil, its regions, and the 27 UF. For Brazil, the detection rate trend and the trend of new cases with G2D decreased, while the proportions of new cases and new multibacillary cases in the elderly age group increased. The result was similar in all the country’s regions and most of the UF.

The decreasing trend in detection rates among the elderly is similar to that of Brazil overall (5). Nery and colleagues (14) indicate that reductions in leprosy detection in endemic municipalities from 2004 – 2011 reflect lower leprosy incidence associated with improved living conditions. This could be attributed, among other factors, to conditional cash transfers by means of the Bolsa Família, a program for families in poverty and extreme poverty. It has three main objectives: to transfer income, to improve access to education and health care, and to offer complementary social programs (15).

Also during the study period, the country registered reductions in the percentage of poor people, from 43.8% to 29.8%; the Gini index, 0.56 to 0.53; the illiteracy rate, 23.1% to 19.6%; and unemployment, 9.0% to 6.9% (15). The socioeconomic determinants of new leprosy case detection is supported by other studies identifying associations between high leprosy rates and poor socioeconomic conditions (16 – 18).

Also important are the influence of leprosy surveillance, decentralization of leprosy control to the primary health care network (19), and the protective effect of BCG vaccination, which ranges from 26% – 61% (20). Multidrug therapy (MDT), despite being widely questioned (21, 22) regarding its ability to reduce bacillary exposure among the population, continues to be a pillar of the Global Leprosy Strategy (1, 3).

**Table 2.** Proportion of new leprosy cases in the elderly in Brazil, its five regions, and 27 *unidades federativas* (UF), 2001-2018

Place	Annual change rate (%)	95%CI^a^		Trend
**North**	**4.2**	**3.6**	**4.9**	**Increasing**
Rondônia	3.4	1.7	5.1	Increasing
Acre	2.9	0.8	5.1	Increasing
Amazonas	3.8	2.7	4.9	Increasing
Roraima	4.3	2.0	6.7	Increasing
Pará	4.2	3.5	5.0	Increasing
Amapá	2.1	0.6	3.5	Increasing
Tocantins	3.7	2.8	4.7	Increasing
**Northeast**	**2.9**	**2.4**	**3.4**	**Increasing**
Maranhão	3.1	2.7	3.5	Increasing
Piauí	2.9	2.4	3.3	Increasing
Ceará	2.6	2.0	3.2	Increasing
Rio Grande do Norte	3.1	1.7	4.5	Increasing
Paraíba	2.4	1.7	3.1	Increasing
Pernambuco	2.8	2.4	3.3	Increasing
Alagoas	3.9	2.5	5.2	Increasing
Sergipe	4.6	3.8	5.4	Increasing
Bahia	3.5	2.8	4.3	Increasing
**Southeast**	**2.8**	**2.5**	**3.1**	**Increasing**
Minas Gerais	2.8	2.2	3.3	Increasing
Espírito Santo	2.8	1.9	3.7	Increasing
Rio de Janeiro	3.0	2.2	3.7	Increasing
São Paulo	2.7	2.3	3.1	Increasing
**South**	**2.7**	**2.1**	**3.2**	**Increasing**
Paraná	2.9	2.3	3.5	Increasing
Santa Catarina	1.4	-0.5	3.3	Stationary
Rio Grande do Sul	2.8	1.9	3.6	Increasing
**Midwest**	**3.2**	**2.9**	**3.6**	**Increasing**
Mato Grosso do Sul	2.4	1.7	3.2	Increasing
Mato Grosso	3.5	3.2	3.8	Increasing
Goiás	3.7	3.1	4.2	Increasing
Federal District	4.5	2.8	6.2	Increasing
**Brazil**	**2.9**	**2.6**	**3.3**	**Increasing**

***Source:*** prepared by the authors based on data from SINAN and Brazilian Institute of Geography and Statistics (IBGE).

a 95%CI: 95% Confidence Interval; Statistical test: Prais-Winsten generalized linear regression

Although the detection rate in the elderly reflects the overall detection rate, since both are decreasing, it reaches much higher levels in the elderly, possibly because this age group is more susceptible. During the study period, the detection rates were about twice as high among the elderly than in the general population (5, 23, 24). The sharper reductions in the South and Southeast compared to the North, Northeast, and Midwest may be related to the higher endemicity levels in these regions where the influence of socioeconomic factors, BCG vaccination, and MDT may lag behind. Seven UF with stationary trends are located in the Northeast and Midwest, corroborating this hypothesis. Moreover, the Northeastern UF showed a statistically lower decline than Southern and Southeastern UF. Associations found between socioeconomic factors and leprosy occurrence reveal that people living in areas with worse poverty (i.e., the Midwest, North, and Northeast) have 5 – 8 times the risk of leprosy (18).

**Table 3.** Proportion of new multibacillary leprosy cases in the elderly in Brazil, its five regions, and 27 *unidades federativas* (UF), 2001-2018

Brazil/regions/UF	Annual change rate (%)	95CI%^a^		Trend
**North**	**1.5**	**1.2**	**1.9**	**Increasing**
Rondônia	1.6	1.0	2.3	Increasing
Acre	1.7	0.4	2.9	Increasing
Amazonas	1.2	0.5	1.9	Increasing
Roraima	1.0	0.4	1.7	Increasing
Pará	1.3	1.1	1.6	Increasing
Amapá	1.7	-0.8	4.2	Stationary
Tocantins	2.4	1.5	3.3	Increasing
**Northeast**	**1.2**	**1.0**	**1.4**	**Increasing**
Maranhão	1.3	1.0	1.5	Increasing
Piauí	2.5	2.0	2.9	Increasing
Ceará	0.5	0.3	0.7	Increasing
Rio Grande do Norte	0.3	-0.7	1.3	Stationary
Paraíba	1.4	0.8	1.9	Increasing
Pernambuco	1.1	0.3	1.9	Increasing
Alagoas	1.3	0.5	2.2	Increasing
Sergipe	1.6	1.0	2.2	Increasing
Bahia	1.8	1.4	2.1	Increasing
**Southeast**	**1.1**	**0.6**	**1.6**	**Increasing**
Minas Gerais	-0.1	-0.8	0.7	Stationary
Espírito Santo	1.8	1.0	2.5	Increasing
Rio de Janeiro	1.1	0.7	1.4	Increasing
São Paulo	2.2	1.9	2.6	Increasing
**South**	**1.4**	**1.2**	**1.7**	**Increasing**
Paraná	1.6	1.3	1.9	Increasing
Santa Catarina	1.6	0.8	2.4	Increasing
Rio Grande do Sul	0.4	-0.1	1.0	Stationary
**Midwest**	**1.8**	**1.5**	**2.1**	**Increasing**
Mato Grosso do Sul	3.0	2.3	3.8	Increasing
Mato Grosso	3.3	2.8	3.8	Increasing
Goiás	0.3	0.1	0.6	Increasing
Federal District	0.8	-0.6	2.1	Stationary
**Brazil**	**1.4**	**1.0**	**1.7**	**Increasing**

***Source:*** prepared by the authors based on data from SINAN and Brazilian Institute of Geography and Statistics (IBGE).

a 95%CI: 95% Confidence Interval; Statistical test: Prais-Winsten generalized linear regression

Higher leprosy incidence in the elderly group may be linked to greater odds of reinfection in endemic areas. Moreover, its long incubation period could lead to late manifestation of signs and symptoms, increasing the possibility of leprosy emerging at older ages (25, 26). Immunosenescence, comorbidities, and malnutrition could also favor the high magnitude of leprosy in this age group (23, 25). Furthermore, the effect of demographic transition, with low mortality and fertility rates, negative growth, a high proportion of elderly people, and increased life expectancy could interfere with reducing detection rate because the denominator becomes larger over time (27).

In 7 UF, detection in the elderly was stationary (Table 1), meaning that leprosy magnitude did not change substantially, despite the growing size of this age group (27). Overall detection in these UF in 2017 reveals different endemicity levels, with medium, high, and hyperendemic rates (28) and a decreasing trend in overall detection in some (24). It may be that despite the downward overall trend and different endemicity levels, greater susceptibility of the elderly favors the emergence of the disease in this group, even in areas with less exposure to leprosy bacilli.

**Table 4.** Rate of new cases with grade 2 physical disability at the time of diagnosis (per 1 000 000 inhabitants) in Brazil, its five regions, and 27 *unidades federativas* (UF), 2001-2018

Brazil/regions/UF	Rate		Annual change rate (%)	95%CI^a^		Trend
	2001	2018				
**North**	**157.9**	**100.0**	**-2.8**	**-4.2**	**-1.3**	**Decreasing**
Rondônia	163.9	108.6	-6.7	-9.8	-3.5	Decreasing
Acre	128.1	116.4	-2.4	-5.6	0.9	Stationary
Amazonas	220.0	32.2	-6.7	-8.6	-4.8	Decreasing
Roraima	451.4	29.5	-9.0	-13.0	-4.9	Decreasing
Pará	131.5	90.9	-2.3	-4.0	-0.5	Decreasing
Amapá	50.9	60.7	-3.7	-8.4	1.2	Stationary
Tocantins	153.0	285.1	2.9	-0.1	6.0	Stationary
**Northeast**	**56.3**	**44.4**	**-1.8**	**-3.4**	**-0.2**	**Decreasing**
Maranhão	186.2	112.8	-1.9	-3.0	-0.7	Decreasing
Piauí	127.8	83.2	-2.0	-4.7	0.9	Stationary
Ceará	68.7	59.0	-2.1	-3.9	-0.3	Decreasing
Rio Grande do Norte	20.1	14.7	-1.7	-8.3	5.5	Stationary
Paraíba	57.9	38.6	-2.2	-5.4	1.2	Stationary
Pernambuco	31.5	36.2	-2.5	-5.3	0.5	Stationary
Alagoas	4.9	26.5	3.7	-4.7	12.8	Stationary
Sergipe	15.3	13.1	3.6	-4.6	12.5	Stationary
Bahia	22.3	25.0	-0.1	-2.3	2.3	Stationary
**Southeast**	**37.8**	**13.3**	**-6.7**	**-7.2**	**-6.3**	**Decreasing**
Minas Gerais	62.1	17.3	-8.4	-10.0	-6.8	Decreasing
Espírito Santo	123.1	13.2	-10.6	-13.1	-8.0	Decreasing
Rio de Janeiro	31.2	12.5	-4.6	-6.2	-3.0	Decreasing
São Paulo	22.5	11.8	-5.1	-6.1	-4.1	Decreasing
**South**	**30.6**	**9.2**	**-7.2**	**-8.9**	**-5.5**	**Decreasing**
Paraná	73.5	13.1	-8.8	-11.0	-6.5	Decreasing
Santa Catarina	9.2	5.2	-5.7	-9.1	-2.1	Stationary
Rio Grande do Sul	6.5	8.1	-2.9	-6.9	1.2	Stationary
**Midwest**	**93.9**	**71.1**	**-2.3**	**-3.2**	**-1.5**	**Decreasing**
Mato Grosso do Sul	62.2	28.0	1.3	-6.7	4.5	Stationary
Mato Grosso	195.2	231.2	0.6	-2.0	3.2	Stationary
Goiás	79.4	44.3	-5.2	-6.1	-4.2	Decreasing
Federal District	52.8	9.1	-9.4	-11.9	-6.8	Decreasing
**Brazil**	**50.6**	**28.3**	**-3.9**	**-4.6**	**-3.2**	**Decreasing**

***Source:*** prepared by the authors based on data from SINAN and Brazilian Institute of Geography and Statistics (IBGE).

a 95%CI: 95% Confidence Interval; Statistical test: Prais-Winsten generalized linear regression

Further supporting this postulation, the proportion of new leprosy cases among the elderly increased throughout Brazil, its regions, and most of the UF despite reductions in overall detection rates during the study period. The rise could be due to the long incubation period of multibacillary leprosy—it can emerge years after the initial infection, which may have occurred when the individual was younger (26). The South and Southeast had the highest proportions of leprosy cases in the elderly to total cases, which corroborates Irgens’ findings (26), given that endemicity in both regions is low (4). On the other hand, the largest increases in this proportion occurred in the North, indicating a possible change in the pattern of endemicity, i.e., switching to the elderly. Although the Northeast had a pattern similar to the South and Southeast, it continued to have lower proportions of cases in elderly. With the exception of Santa Catarina, there was an increase in the proportion of the elderly with leprosy in all the UF, although without significant differences among them.

The proportion of new multibacillary leprosy cases in elderly also showed an increasing trend for Brazil, its regions, and most of its UF. Nonetheless, rates were more stable, increasing only about 1% a year, with narrow confidence intervals and without differences among regions. The more homogenous pattern within Brazil’s heterogeneous epidemiologic context of leprosy could be more closely linked to the population’s increased life expectancy than to changes in endemicity. Individuals with a previous infection could have more time to develop the leprosy, which in this later stage of life, would be predominantly multibacillary.

Aging comes with an increased risk of developing biological, socioeconomic, and/or psychosocial vulnerabilities due to declining senescence. These vulnerabilities reduce capacity for self-protection (29), and any physical disability arising from leprosy would further threaten an elderly individual.

This study found a decreasing trend in detection rates for cases of leprosy with G2D in the elderly in Brazil, its regions, and 13 UF. However, in 2018 this indicator reached 28.3/1 million, whereas for Brazil’s general population, it was 10/1 million (4). This illustrates the challenge that Brazil faces in reaching the target of < 1/1 million, established by the Global Leprosy Strategy 2016 – 2020 (3). In addition, 14 UF had a stationary trend, therefore the rates continued to be high over the time series, possibly due to delayed diagnosis (3).

With regard to declines in detection and G2D rates, the latter indicator was more heterogeneous across the UF, with 13 having a stationary trend. The detection rate trend was stationary in 7 UF. Differences were also found among the regions. UFs with decreasing detection rates were in the Southeast, and those with stationary G2D rate trends were in the North, Northeast, South, and Midwest.

Leprosy can mimic dermatological, rheumatological, and neurological diseases. Diabetic neuropathy and rheumatoid arthritis, which are also prevalent in the elderly, may hinder differential diagnosis and favor delayed treatment (30). Moreover, elderly people with disabilities have greater difficulty accessing health services (31). A higher risk of physical disability in the elderly is possibly multifactorial and related to biological issues, comorbidities, and difficulties with diagnosis and access to health services. Thus, it is essential that the Health Care Model for the Elderly and the process of qualifying care include leprosy within the scope of multi-professional health team activities, including active case tracing, contact investigation, health education, and functional assessment (7).

Although Brazilian legislation on the care of the elderly is comprehensive and advanced, in practical terms it falls far short of overcoming the social vulnerabilities to which some are exposed (13). The results of this study corroborate this worrying situation and the leprosy mortality trends (32) that show the elderly to be at far greater risk than other age groups in the North and Northeast regions of Brazil (RR 7.9; *P* value < 0.0001) .

Leprosy control among the elderly is important for achieving the Global Leprosy Strategy 2016 – 2020 targets (3). The increasing proportions of new cases and multibacillary cases among the elderly reinforce the need for specific active tracing and contact investigations in this age group (5)—especially the multibacillary leprosy cases, which are responsible for bacillus transmission (33). Also, elderly people with leprosy should be regarded as having both a communicable disease and a chronic condition, in accordance with the “Guidelines for SUS health care for the elderly” (7).

### Limitations.

This study had limitations inherent to ecological studies, which cannot establish a relationship between cause and effect. Of note is the complexity of leprosy evolution, with its long incubation periods, possibility of misclassification, and attributing G2D. There were also limitations caused by changes to SINAN, which may have impacted data quality, such as under- or over-reporting (17, 24). Furthermore, a decline in detection rates can be influenced by operational issues (21), such as accurate disease diagnosis; community awareness (3, 15); differences in coverage and access to comprehensive health care; and operational interventions in program management (34).

Despite these limitations, ecological studies enable hypotheses to be formed and can support decision making (17). Regarding data quality, detection rates are a function of true incidence and a health system’s diagnostic capacity since surveillance systems can vary operationally (19). Notwithstanding, case detection trends can be considered close to incidence when the indicator of new cases with G2D remains stable over years (24, 28, 33).

## Conclusions

Detection rates of new leprosy cases in elderly people in Brazil are decreasing, while the proportion of new cases and multibacillary cases are trending upwards. This context indicates that new case detection is shifting to older age groups, and factors related to demographic transition and immunosenescence are having an influence. The inadequate reduction of G2D rates indicates that a higher risk of physical disability persists among the elderly despite declining detection. Specific and active tracing and contact investigation are needed in this age group.

## Author contributions.

MCNR, MLN, and LPG collaborated on the project concepts, data analysis, data interpretation, drafting of the paper, and revisions. All authors reviewed and approved the final version.

## Acknowledgements.

The authors wish to thank Elaine da Rós Oliveira and Mábia Milhomem Bastos for collaborating on map development and Vera Lúcia Gomes de Andrade for discussions on the topic.

## Disclaimer.

Authors hold sole responsibility for the views expressed in the manuscript, which may not necessarily reflect the opinion or policy of the *RPSP/PAJPH* and/or PAHO.
